# Unravelling parasitic spinal infections: a narrative review on diagnosis, treatment strategies, and collaborative management approaches

**DOI:** 10.3205/dgkh000662

**Published:** 2026-07-09

**Authors:** Anand Kumar Das, Sona Bhardwaj, Rijhul Lahariya, Mainak Sinha, Saraj Kumar Singh, Simmi Kishore

**Affiliations:** 1 Neurosurgery, All India Institute of Medical Sciences, Patna, Bihar, India; 2 Department of Microbiology, Netaji Subhas Medical College & Hospital (NSMCH), Amhara, Bihta, Patna, Bihar, India; 3 MBBS, All India Institute of Medical Sciences, Patna, Bihar, India; 4 Department of Anaesthesiology and Critical Care Medicine, Indira Gandhi Institute of Medical Sciences, Patna, Bihar, India

**Keywords:** parasitic infections, spinal infection, toxocariasis, neurocysticercosis, echinococcosis

## Abstract

**Introduction:**

Parasitic infections are more common in low hygiene regions but can occur in developed countries. They often cause significant morbidity and death, especially in the central nervous system. Some spinal infections are caused by *Taenia solium*, which causes spinal neurocysticercosis. *Echinococcus*, which causes spinal echinococcosis, frequently infects the lumbar and thoracic spines. Spinal cord schistosomiasis caused by *Schistosoma* infection has serious clinical consequences. Toxoplasmosis is common in immunocompromised patients, whereas Toxocariasis is generally more common as a childhood infection, caused by ingestion of the eggs.

This review article examines parasitic spinal infections, patient demographics, afflicted spinal regions, medical and surgical treatments, follow-up durations, and results.

**Method:**

The search strategy included (Parasitic) OR (Neurocysticercosis) OR (Echinococcosis) OR (Sparganosis) OR (Schistosomiasis) OR (Gnathostomiasis) OR (Lagochilascariasis) OR (Toxocariasis) OR (Toxoplasma)) AND ((Spine) OR (Spinal)) AND (Infection).

**Results:**

35 articles with 44 participants (average age of 40.1±12.6 years) were included. Most affected were the thoracic (50%) and lumbar (15.9%) spines. Neurocysticercosis was the most common infection (45.5%), followed by echinococcosis (31.8%), sparganosis (6.8%), schistosomiasis (4.5%), gnathostomiasis (4.5%), and single occurrences of lagochilascariasis and toxocariasis (2.3% each).

Albendazole was administered in 90.9% of cases. Depending on the situation, 35 out of 44 (79.5%) of cases required surgery, which could involve fixation or not. All patients survived, with an average follow-up of 18.6±24.8 months.

Neuroimaging, serology, and clinical symptoms determined the diagnosis. Antiparasitic and anti-inflammatory medicines are utilised, and surgery or spinal cord decompression may be explored for unclear diagnoses.

## Zusammenfassung

**Einleitung:**

Parasitäre Infektionen treten häufiger in Regionen mit schlechten hygienischen Verhältnissen auf, können aber auch in Industrieländern vorkommen. Sie verursachen oft erhebliche Morbidität und Todesfälle, insbesondere im Zentralnervensystem. Einige Wirbelsäuleninfektionen werden durch Taenia solium verursacht, der eine spinale Neurozystizerkose hervorruft. Echinococcus, der eine spinale Echinokokkose verursacht, befällt häufig die Lenden- und Brustwirbelsäule. Die durch Schistosomen verursachte Schistosomiasis des Rückenmarks hat schwerwiegende klinische Folgen. Toxoplasmose tritt häufig bei immungeschwächten Patienten auf, während Toxokariasis in der Regel eher als Infektion im Kindesalter auftritt, die durch die Aufnahme von Eiern verursacht wird.

Die Übersicht befasst sich mit parasitären Wirbelsäuleninfektionen, demografischen Daten der Patienten, betroffenen Wirbelsäulenregionen, medizinischen und chirurgischen Behandlungen, Nachbeobachtungszeiträumen und Ergebnissen.

**Methode:**

Die Suchstrategie beinhaltete (Parasitär) ODER (Neurozystizerkose) ODER (Echinokokkose) ODER (Sparganose) ODER (Schistosomiasis) ODER (Gnathostomiasis) ODER (Lagochilascariasis) ODER (Toxokariasis) ODER (Toxoplasma)) UND ((Wirbelsäule) ODER (Spinal)) UND (Infektion).

**Ergebnisse:**

Es wurden 35 Artikel mit 44 Teilnehmern (Durchschnittsalter 40,1 ± 12,6 Jahre) einbezogen. Am häufigsten betroffen waren die Brustwirbelsäule (50 %) und die Lendenwirbelsäule (15,9%). Am stärksten betroffen waren die Brustwirbelsäule (50%) und die Lendenwirbelsäule (15,9%). Neurozystizerkose war die häufigste Infektion (45,5%), gefolgt von Echinokokkose (31,8%), Sparganose (6,8%), Schistosomiasis (4,5%), Gnathostomiasis (4,5%) und vereinzelten Fällen von Lagochilascariasis und Toxokariasis (jeweils 2,3%).

In 90,9% der Fälle wurde Albendazol verabreicht. Je nach Situation mussten 35 von 44 Fällen (79,5%) operiert werden, wobei das mit oder ohne Fixierung erfolgen konnte. Alle Patienten überlebten bei einer durchschnittlichen Nachbeobachtungszeit von 18,6 ± 24,8 Monaten.

Die Diagnose wurde anhand von Neuroimaging, Serologie und klinischen Symptomen gestellt. Es wurden antiparasitäre und entzündungshemmende Medikamente eingesetzt und bei unklaren Diagnosen können auch ein chirurgischer Eingriff oder eine Rückenmarksdekompression in Betracht gezogen werden.

## Introduction

Parasitic infections are more common in areas with poor hygiene, but they can also occur in developed countries [1]. They carry high morbidity and mortality, particularly when involving the central nervous system (CNS) [1]. Several parasite species are capable of invading the CNS, often leading to severe and life-threatening neurological disease [2]. Infection typically follows ingestion of parasite eggs, which develop through larval stages, reach the bloodstream, and disseminate systemically. They can be transmitted via contaminated food or water, direct skin penetration by infective larvae, vector-borne transmission through insect bites, and occasionally through contact with contaminated soil or surfaces [3]. While the host immune system can often control these infections, some parasites evade immune detection and persist [1]. Early recognition and intervention are essential to limit neurological damage and improve outcomes. 

Among endoparasites, protozoa and helminths are of particular clinical relevance. Spinal neurocysticercosis, caused by Taenia solium, represents the most common parasitic infection of the spine, accounting for approximately 2.7% of all neurocysticercosis cases [1]. Spinal echinococcosis, due to Echinococcus species, is rarer but primarily affects the thoracic spine, followed by the lumbar region [1]. Spinal cord schistosomiasis is a recognized though uncommon manifestation of Schistosoma infection, often resulting in serious neurological sequelae [3]. Toxocariasis, typically a pediatric disease, arises from ingestion of parasite eggs [4]. Toxoplasma gondii infection (toxoplasmosis) is more commonly seen in immunocompromised individuals.

Spinal infections may be classified according to the anatomical structures involved, including the vertebral bodies, intervertebral discs, and the spinal canal [5]. Among spinal regions, the thoracic and lumbar segments are most frequently affected. This review examines global cases of spinal parasitic infections, focusing on patient demographics, anatomical involvement, treatment strategies, follow-up duration, and clinical outcomes. 

## Method

This literature review included all articles indexed in the National Library of Medicine PubMed from 2014 to 2024. The search strategy employed the following syntax:

((Parasitic) OR (Neurocysticercosis) OR (Echinococcosis) OR (Sparganosis) OR (Schistosomiasis) OR (Gnathostomiasis) OR (Lagochilascariasis) OR (Toxocariasis) OR (Toxoplasma)) AND ((Spine) OR (Spinal)) AND (Infection). 

 All types of studies reporting parasitic spinal infections from 2014 to 2024, related to any segment of the spine, in English literature, were included, considering patients of above 18 age with no restriction on gender and no restrictions related to any parasitic disease, follow-up duration, or spinal segment. Exclusion criterias were no defined. 

Relevant clinical and demographic data from each article were extracted, compiled, and applied descriptive statistical methods. Variables included patient age, gender, involved spinal region, definitive diagnosis, medical therapy administered, surgical intervention (if any), follow-up duration, and clinical outcome (alive or deceased). For medical therapy, details of antiparasitic agents used were recorded. For surgical management, interventions as either with fixation or without fixation were categorized, in the last with classification of the approach as curettage, drainage, decompression, or laminectomy when specified. Outcome parameters included both mortality and recovery status at the last follow-up. 

## Results

After applying inclusion criteria, we included 35 articles. A total of 44 patients were included with a mean age of 40.1±12.6 years (range: 21–64 years) [1], [3], [4], [5], [6], [7], [8], [9], [10], [11], [12], [13], [14], [15], [16], [17], [18], [19], [20], [21], [22], [23], [24], [25], [26], [27], [28], [29], [30], [31], [32], [33], [34], [35], [36] (Table 1 (Tab. 1)). Among the study participants, 28 were male (63.6%) (Table 1 (Tab. 1)). 

The distribution of spinal involvement showed the thoracic region to be the most commonly affected (50%), followed by the lumbar spine (15.9%) and the lumbosacral region (L1–S2, 13.7%). Additionally, there were 3 cases (6.9%) involving the thoraco-cervical region (C6–T9), and 2 cases (4.5%) each involving the cervical spine, thoracolumbar spine (T10–L5), and whole spine (C7–L5). “Whole spine” involvement was defined as infection affecting nearly all spinal segments (Table 1 (Tab. 1)). 

Neurocysticercosis was the most prevalent diagnosis, observed in 45.5% of cases, followed by echinococcosis (31.8%), sparganosis (6.8%), schistosomiasis (4.5%), gnathostomiasis (4.5%), and single cases (2.3% each) of lagochilascariasis, toxoplasmosis, and toxocariasis (Table 1 (Tab. 1)).

Albendazole was the most frequently administered antiparasitic agent, prescribed in 90.9% of cases, including those with neurocysticercosis, echinococcosis, gnathostomiasis, lagochilascariasis, and toxocariasis. Praziquantel was used in 9.1% of cases, specifically in sparganosis and schistosomiasis (Table 2 (Tab. 2)). 

Surgical intervention was required in most cases. Among these, 20% of patients—primarily those with echinococcosis and toxocariasis—underwent surgery with spinal fixation. The remaining 80% underwent surgery without fixation, which included decompression, debridement, and abscess drainage (Table 2 (Tab. 2)). Indications for fixation included multi-vertebral involvement, compressive radiculopathy or myelopathy, prior spinal instrumentation, or pre-existing spinal deformities (e.g., kyphoscoliosis). Echinococcosis was the most common indication for surgical fixation in this cohort. Of the 44 patients with reported clinical outcomes, all (100%) were alive at final follow-up. The mean follow-up duration was 18.6±24.8 months (Table 2 (Tab. 2)). 

## Discussion

The review identified neurocysticercosis (NCC) as the most common parasitic spinal infection globally. NCC is caused by the larval cyst *Cysticercus cellulosae* of *Taenia solium*. Transmission occurs either through ingestion of eggs shed in the faeces of human tapeworm carriers or by consuming undercooked pork containing cysticerci, the infective larval stage. Once ingested, eggs hatch into larvae that penetrate the intestinal wall and disseminate hematogenously to the central nervous system, including the spine, where they develop into cysticerci causing inflammation and neurological symptoms [1]. Hydatid disease, or cystic echinococcosis, emerged as the second most prevalent spinal parasitic infection, resulting from the larval form of *Echinococcus granulosus* [5]. Sparganosis ranked third in frequency, with three reported cases. Additionally, we found two cases of spinal schistosomiasis, an acute and chronic parasitic disease caused by blood flukes of the genus *Schistosoma* [31]. Two cases of gnathostomiasis were also identified. Rarer spinal parasitic infections included single cases of lagochilascariasis, toxoplasmosis, and toxocariasis. These findings reflect the diverse spectrum of parasitic agents capable of spinal involvement and emphasize the importance of considering these differential diagnoses in endemic areas.

### Clinical presentation

Based on the review, the mean age of presentation for parasitic spinal infections was 40.1±12.6 years, with a clear male predominance (63.6%). The thoracic spine was the most frequently involved region, followed by the lumbar and lumbosacral segments. 

Spinal neurocysticercosis commonly presents with neurological deficits such as radiculopathy, myelopathy, or cauda equina syndrome, depending on the location and extent of cysticerci involvement. These deficits manifest as limb weakness, sensory loss, and sphincter disturbances. Meningitis associated with spinal NCC is typically leptomeningitis, involving inflammation of the pia and arachnoid mater, and can affect both the cranial and spinal meninges [13], [19]. 

Echinococcosis typically affects the liver and lungs, with CNS involvement being relatively uncommon [37]. In spinal cases, symptoms depend on the extent of cord compression and may include localized pain and sensory disturbances [32].

Sparganosis, though infrequently affecting the spine, primarily involves the lumbosacral region and presents with back pain, radiculopathy, neurogenic bladder, and paraparesis [2], [5]. 

Schistosomiasis-associated myelopathy typically manifests as transverse myelitis due to spinal cord necrosis [2]. Symptom onset includes lower back pain and limb weakness, peaking approximately 15 days after initial presentation; the thoracolumbar region is most commonly affected [2]. 

Gnathostomiasis invades the spinal cord via nerve roots, initially causing radicular pain and headache, followed by progressive motor deficits ranging from mono- to quadriparesis [1].

Spinal lagochilascariasis may present with localized pain, stiffness, and progressive neurological deficits due to compression of neural structures by parasitic granulomas [38]. This may lead to signs of cord compression including weakness, sensory loss, and autonomic dysfunction [38]. 

Spinal toxoplasmosis typically occurs in immunocompromised individuals and manifests with paraparesis, bilateral sensory impairment, urinary retention, localized pain, fever, brisk deep tendon reflexes, and extensor plantar response. Toxocariasis may result in motor and sensory deficits, predominantly affecting the lower extremities [5]. 

These findings underscore the heterogeneous clinical presentations of spinal parasitic infections, often mimicking other etiologies of myelopathy and radiculopathy, thereby necessitating high clinical suspicion in endemic regions. 

### Diagnosis

The 2017 revised diagnostic criteria for neurocysticercosis emphasize neuroimaging findings in conjunction with clinical exposure history [2]. Both, computed tomography (CT) and magnetic resonance imaging (MRI), are essential for characterizing lesion morphology, stage, and anatomical localization [2]. Post-imaging, serological confirmation is critical—enzyme-linked immunoelectrotransfer blot (EITB) remains the gold standard, with approximately 98% sensitivity and near 100% specificity in cases with multiple brain cysts. 

In cystic echinococcosis, epidemiological exposure—particularly contact with sheepdogs in endemic areas—and the presence of cyst-like lesions – raise suspicion [5]. Imaging modalities such as CT, MRI, and ultrasonography are central to diagnosis. Plain radiographs may reveal cystic or irregular bony erosions, while CT differentiates hydatid cysts from other lytic processes [2]. MRI typically demonstrates a multilocular, spherical, thin-walled lesion without septations—features that can mimic neoplastic or granulomatous diseases, necessitating detailed radiologic-pathologic correlation [2].

Cerebral sparganosis mansoni poses diagnostic challenges due to nonspecific clinical and radiologic findings [14], [18]. ELISA detecting anti-sparganum antibodies in cerebrospinal fluid (CSF) or serum provides high diagnostic accuracy [14]. 

Schistosomiasis requires evidence of active infection; although histopathology is definitive, its invasiveness limits utility [39]. Diagnosis is thus usually inferred from clinical presentation, positive serology or stool tests, and suggestive neuroimaging or CSF findings [39]. 

Gnathostomiasis is suspected based on the triad of eosinophilia, migratory symptoms, and exposure history [15]. Larval recovery remains definitive, while immunoblotting serves as the primary serological tool. MRI can visualize migratory tracks of larvae [10], [15].

Lagochilascariasis diagnosis relies on histopathological identification from biopsied tissue; no validated serological assays currently exist [38]. 

Toxoplasmosis diagnosis, particularly in immunocompromised individuals (CD4<100/mm³), is based on T. gondii IgG seropositivity, absence of prophylaxis, and characteristic ring-enhancing lesions on imaging. In toxocariasis, diagnosis integrates clinical features with serological evidence via ELISA for anti-Toxocara antibodies [3]; findings such as eosinophilia and hypergammaglobulinemia provide supportive evidence [3]. 

Overall, a multimodal diagnostic approach combining clinical, radiological, and serological parameters is essential for accurate identification.

### Management

Management of neurocysticercosis primarily involves antiparasitic therapy, albendazole (15 mg/kg/day in two divided doses for 10–14 days) and/or praziquantel combined with corticosteroids to control inflammation [37]. 

Surgical intervention is reserved for cases with multilevel vertebral involvement, compressive myelopathy, or spinal deformities such as kyphosis. Procedures without fixation—decompression, debridement, and abscess drainage—are common, while fixation is indicated when spinal stability is compromised. According to the American Society for Microbiology’s consensus guidelines, laminectomy with cyst removal remains the mainstay surgical approach [37]. 

In our review, all 20 neurocysticercosis cases received albendazole, with 15 undergoing surgeries without fixation, 1 requiring fixation, and 3 unreported. Although early diagnosis and effective medical therapy have reduced surgical necessity, operative management may be required for diagnostic confirmation or spinal cord decompression [5]. Postoperative risks include inflammation and arachnoiditis, necessitating careful patient selection and monitoring [5].

For spinal echinococcosis, albendazole therapy is recommended for 6 to 12 months to prevent cyst recurrence [6], [32]. In our review, all 14 reported patients received albendazole at 15 mg/kg daily in two divided doses for 4 weeks postoperatively. Surgical management, primarily via laminectomy, remains the definitive treatment aimed at complete cyst excision and decompression of the spinal cord [32]. Among 12 cases with surgical data, 5 underwent surgery without fixation, 8 required fixations, and 1 case had no reported surgical intervention.

For spinal sparganosis, high-dose praziquantel (25 mg/kg thrice daily) is effective, particularly in inoperable cases [5], [14]. In our review of 3 cases, two were treated medically with praziquantel and one with albendazole. Surgical removal of granulation tissue and parasites remains essential, especially when lesions are densely adherent to nerve roots in the subarachnoid space [5]. All cases in our series underwent surgery without fixation, limited to decompression and laminectomy.

In spinal schistosomiasis, high-dose corticosteroid therapy is administered to control inflammation, typically methylprednisolone 1.0 g IV daily for five days, followed by oral prednisone (1 mg/kg/day) tapered over six months to improve neurological outcomes [17]. Praziquantel, effective against all Schistosoma species, is given as a single oral dose of 50 mg/kg [39]. For acute schistosomiasis, Artemether—an antimalarial—can target immature Schistosoma larvae during the first three weeks of infection [39]. In cases with significant inflammation, corticosteroids alone may suffice [39]. In our review, both reported cases received praziquantel; however, neither documented surgical intervention. Surgical options, including mass excision, decompressive laminectomy, and nerve root decompression, may be considered as alternatives to spinal fixation in appropriate cases [39].

Historically, surgical excision was the only treatment for gnathostomiasis; however, recent studies have demonstrated albendazole’s efficacy, with some reports supporting sequential albendazole and ivermectin (150–250 µg/kg) therapy [1]. In our review, two cases received albendazole, with only one undergoing decompression surgery without fixation. For lagochilascariasis, a single case was treated with albendazole (400 mg once), and abscess drainage was performed surgically without fixation [40].

Spinal toxoplasmosis was managed medically with anti-toxoplasma therapy (trimethoprim-sulfamethoxazole) alongside antiretroviral therapy (zidovudine, lamivudine, efavirenz), obviating the need for surgery. Albendazole remains the preferred treatment for spinal toxocariasis due to its low toxicity, excellent CNS penetration, and high serum concentrations, often combined with corticosteroids to mitigate inflammation and immunologic effects [8]. Our review included one toxocariasis case treated with albendazole and surgical fixation.

### Prognosis

Follow-up in parasitic spinal infections was primarily conducted through symptom assessment and physical examination, with MRI performed in select cases [13], [14]. 

The prognosis of spinal neurocysticercosis depends on factors including lesion location, symptom duration, and inflammation extent [7]. In our review, all patients survived post-treatment with a mean follow-up of 29.3±31.5 months. 

Similarly, spinal echinococcosis patients demonstrated good outcomes, with a mean follow-up of 10.6±7.9 months and all alive after therapy. 

Spinal sparganosis prognosis ranges from fair to good, consistent with our findings where all three cases were alive at a mean follow-up of 12.3±11.5 months [5]. 

Early diagnosis and treatment of schistosomiasis correlate with better outcomes; our two cases survived with a mean follow-up of 6 months [3]. 

The single reported gnathostomiasis case survived with a follow-up of 3.25±3.89 months. 

One case of lagochilascariasis survived with a 2.5-month follow-up. 

For toxoplasmosis, follow-up was 1 month for the single case, while no literature suggests recurrence after surgical management of spinal toxocariasis; our case was alive with a 2-month follow-up [5].

### Prevention strategy

Prevention of certain parasitic spinal infections is critical to improving patient outcomes. For gnathostomiasis, proper cooking of meat is essential to inactivate larvae and prevent infection [1]. Public health education aimed at modifying eating habits plays a vital role in reducing incidence and improving prognosis [1]. Following effective treatment of lagochilascariasis, vigilant monitoring for potential recurrence is necessary due to the risk of relapse [40]. Vaccination of sheep with an *E. granulosus* recombinant antigen (EG95) offers encouraging prospects for prevention and control. The vaccine is currently being produced commercially and is registered in China and Argentina. Trials in Argentina demonstrated the added value of vaccinating sheep, and in China the vaccine is being used extensively [2]. 

Looking ahead, integrating data-driven approaches may enhance infection control further. Machine learning models hold promise in predicting disease severity and mortality, enabling earlier and more tailored interventions [41]. Additionally, shifting toward pathogen-directed therapy based on local resistance patterns—rather than relying on empiric broad-spectrum antimicrobials—combined with the development of targeted antimicrobial stewardship programs, offers a more sustainable and effective approach to long-term prevention and management [42].

## Conclusion

Parasitic spinal infections, though rare, can cause serious neurological deficits. Neurocysticercosis (45.5%) and echinococcosis (31.8%) were most common, often affecting the thoracic spine (50%) and males (63.6%). Symptoms included pain and weakness. Diagnosis used imaging, serology, and histopathology. Albendazole was the main treatment (90.9%), with surgery needed in 79.5% for decompression and drainage; fixation was used in 20%. All patients survived, highlighting the value of early diagnosis. Future studies should define treatment protocols, assess long-term outcomes, and improve early detection in endemic areas.

## Notes

### Authors’ ORCIDs

- Das AK: https://orcid.org/0000-0002-0705-9393
- Lahariya R: https://orcid.org/0009-0003-5769-4509
- Sinha M: https://orcid.org/0000-0002-2286-0701
- Singh SK: https://orcid.org/ 0000-0003-3156-7096

### Funding

None. 

### Acknowledgments

The authors acknowledge all consultants in the department of Neurosurgery for their guidance and assistance.

### Author contribution

Anand Kumar Das and Sona Bhardwaj contributed equally. 

### Competing interests

The authors declare that they have no competing interests.

## Figures and Tables

**Table 1:**
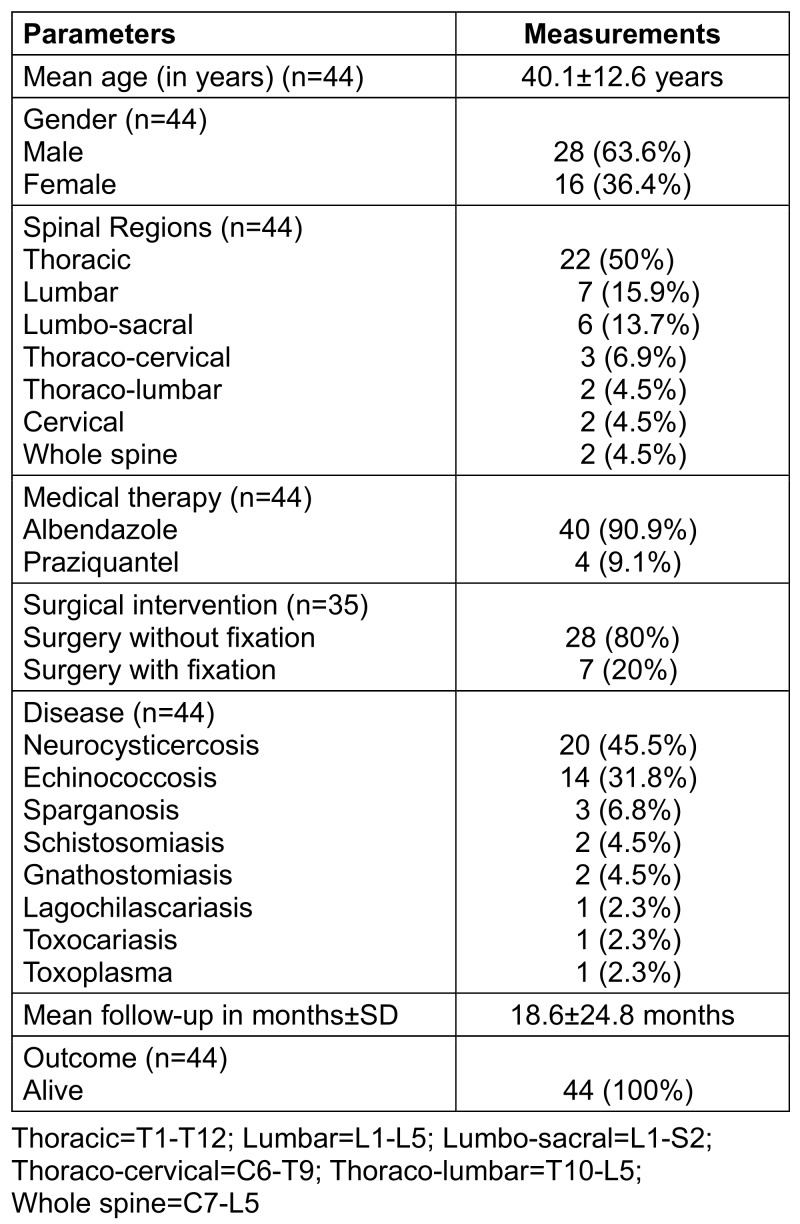
Summary of the patients included in our study

**Table 2:**
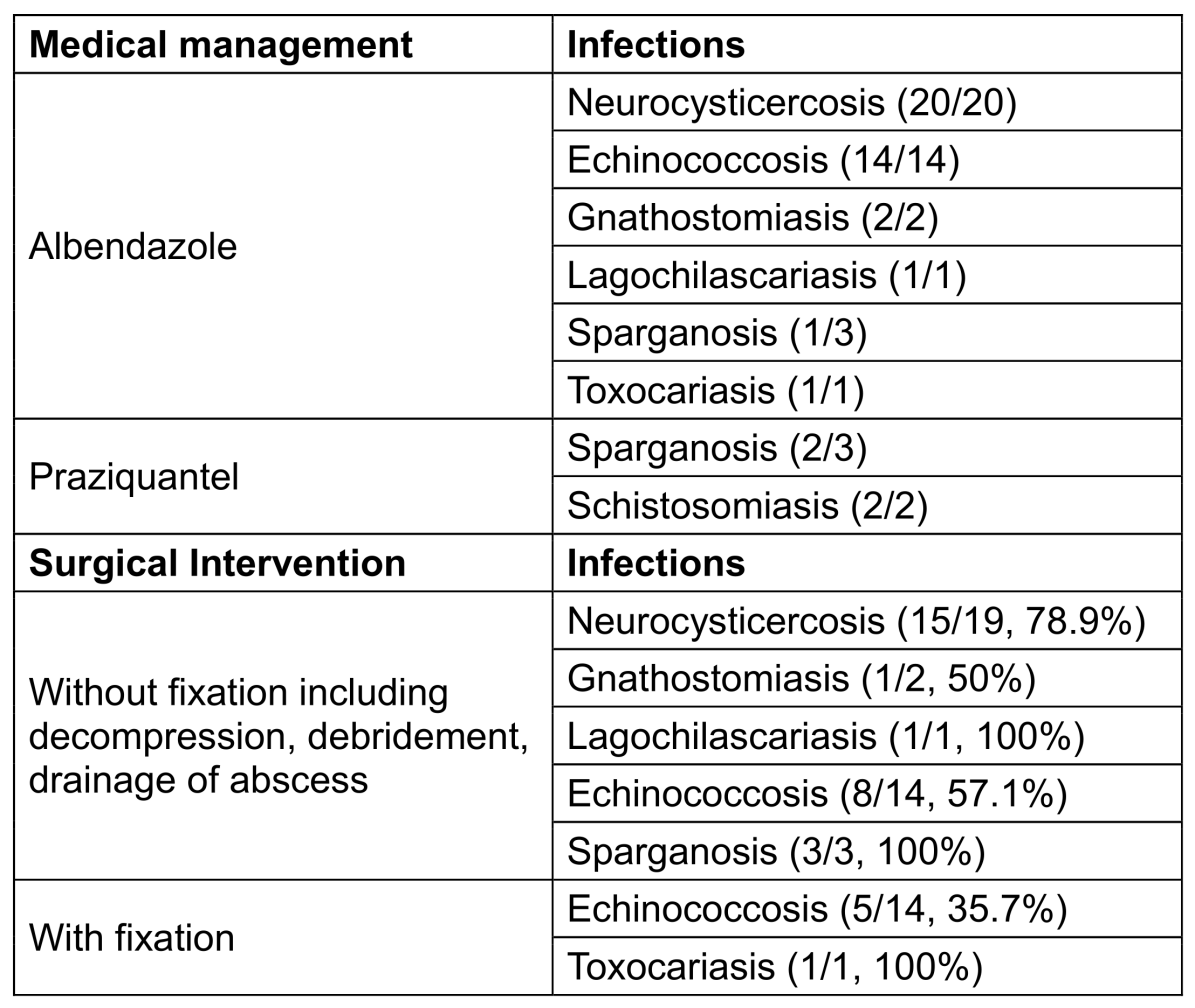
Medical and surgical management in infections
